# Pretreatment with Human Lactoferrin Had a Positive Effect on the Dynamics of Mouse Nigrostriatal System Recovery after Acute MPTP Exposure

**DOI:** 10.3390/biology10010024

**Published:** 2021-01-01

**Authors:** Marina Yu. Kopaeva, Anton B. Cherepov, Mikhail V. Nesterenko, Irina Yu. Zarayskaya

**Affiliations:** 1 National Research Center «Kurchatov Institute», 1 Akademika Kurchatova sq., 123182 Moscow, Russia; ipmagus@mail.ru (A.B.C.); irzar2003@mail.ru (I.Y.Z.); 2 «Lactobio» LLC, 29 Prospekt Vernadskogo, 119331 Moscow, Russia; mnester2000@mail.ru

**Keywords:** human lactoferrin, Parkinson’s disease, MPTP, substantia nigra, striatum, dopaminergic neurons

## Abstract

**Simple Summary:**

Parkinson’s disease (PD) is the progressive neurodegenerative disorder characterized by the degeneration of dopaminergic neurons in substantia nigra and depletion of dopamine in the striatum. Many efforts are now focused on the search for agents that can weaken PD progression, prevent further neurodegeneration, and restore the degenerated dopaminergic neurons. The present study explores the effects of lactoferrin (a multifunctional protein from the transferrin family) in the MPTP-treated mice as the model of dopaminergic neuron loss. MPTP is a neurotoxin that upon systemic administration selectively destroys dopaminergic neurons of the nigrostriatal system inducing number of the symptoms observed in idiopathic PD. Our data suggest that pretreatment with human lactoferrin significantly alleviated MPTP toxicity. This was manifested in improved motor functions and exploratory behavior, partial recovery of the number of tyrosine hydroxylase (TH)-positive cells in the substantia nigra, and TH-positive fibers in the striatum. The results of this work elucidate the role of lactoferrin in protective and compensatory mechanisms and provide the basis for potential use of this protein in the treatment of human neurodegenerative diseases.

**Abstract:**

We studied the effect of human lactoferrin (hLf) on degenerative changes in the nigrostriatal system and associated behavioral deficits in the animal model of Parkinson disease. Nigrostriatal dopaminergic injury was induced by single administration of 1-methyl-4-phenyl-1,2,3,6-tetrahydropyridine (MPTP; 40 mg/kg) to five-month-old C57Bl/6 mice. Behavioral disturbances were assessed in the open field and rotarod tests and by the stride length analysis. Structural deficits were assessed by the counts of tyrosine hydroxylase (TH)-immunoreactive neurons in the substantia nigra and optical density (OD) of TH-immunolabeled fibers in the striatum. Acute MPTP treatment induced long-term behavioral deficit and degenerative changes in the nigrostriatal system. Pretreatment with hLf prevented body weight loss and promoted recovery of motor functions and exploratory behavior. Importantly, OD of TH-positive fibers in the striatum of mice treated with hLf almost returned to normal, and the number of TH-positive cells in the substantia nigra significantly increased on day 28. These results indicate that hLf produces a neuroprotective effect and probably stimulates neuroregeneration under conditions of MPTP toxicity in our model. A relationship between behavioral deficits and nigrostriatal system disturbances at delayed terms after MPTP administration was found.

## 1. Introduction

Parkinson’s disease (PD), a progressive neurodegenerative disorder, is characterized by the degeneration of dopaminergic neurons in substantia nigra pars compacta (SNpc) and resultant depletion of dopamine (DA) in the striatum, leading to clinical symptoms of rigidity, tremor, and bradykinesia [1]. The pathogenesis of PD is not completely understood; however, oxidative stress is one of the popular hypotheses. Previous studies have demonstrated that excessive generation of reactive oxygen species (ROS), oxidative stress, neuroinflammation, and mitochondrial dysfunction can lead to the loss of dopaminergic neurons [2,3,4,5]. Many efforts are now focused on the search for agents that can weaken PD progression, prevent further neurodegeneration, and restore the degenerated dopaminergic neurons.

MPTP (1-methyl-4-phenyl-1,2,3,6-tetrahydropyridine)-induced neurodegeneration in mice is one of the most commonly used animal models of PD [6,7]. MPTP is a neurotoxin that upon systemic administration crosses the blood–brain barrier and selectively destroys dopaminergic neurons of the nigrostriatal system inducing a number of the symptoms observed in idiopathic PD [7,8]. However, this mouse model is limited by rapid development of toxicosis, while the idiopathic form of PD progresses slowly, and its inability to demonstrate persistent motor deficits. Its toxicity is determined by its active metabolite 1-methyl-4-phenylpyridium ion (MPP+) that is transported into neurons by the dopamine reuptake system and disturbs mitochondrial respiratory processes [9,10]. This metabolite also enhances lipid peroxidation, a process triggered by the overproduction of free radicals [11,12].

Previous research has demonstrated iron accumulation in the brain of PD patients [13,14] and MPTP-treated mice [15,16]. Increased iron concentrations can induce oxidative stress through ROS generation and cause neuronal damage [15,17,18].

Lactoferrin (Lf) is a multifunctional protein from the transferrin family characterized by high affinity for Fe^3+^. It is present in various secretions of mammals, such as milk, saliva, tears, nasal fluids, and in neutrophilic leukocytes [19,20]. This protein is involved in many physiological functions, including regulation of iron absorption and immune responses. It demonstrates antibacterial, antiviral, anti-inflammation, anticarcinogenic, antioxidant, and radioprotective activities [21,22,23,24]. Neurons of PD patients have been found to accumulate high concentrations of Lf [25]; on the other hand, the number of Lf receptors is increased in dopaminergic neurons resistant to the disease process [26]. This prompts studying the potential of lactoferrin in correction of neurodegeneration. Administration of exogenous Lf can improve neuroprotection, neurodevelopment, and learning behavior in mammals by altering the expression profiles of genes involved in various neuronal signaling pathways [27,28]. It was demonstrated that Lf can cross the blood–brain barrier via receptor-mediated transcytosis [29,30]. Previously, we have shown that the exogenous Lf was transferred into the brain after intranasal, sublingual, and intraperitoneal administration. Highly specific binding of human Lf was found in the nuclei of neurons, astrocytes, and microglial cells [31].

The aim of the presented research was to investigate the effects of human Lf (hLf) in the MPTP-treated mice as the model of dopaminergic neurons loss. We assessed the time course of degenerative changes in the nigrostriatal system and the associated behavioral consequences of acute MPTP administration. The relationship between the neuronal degeneration and animal behavior was explored. Functional impairment was estimated using the rotarod and open field tests and stride length analysis. Structural deficits were assessed by the counts of tyrosine hydroxylase (TH)-positive cells in the SNpc and the density of TH-positive fibers in the striatum.

## 2. Materials and Methods

### 2.1. Animals and Treatments

Thirty-eight five-month-old C57Bl/6 male mice (27–35 g) used in this study were obtained from the Branch of the Shemyakin and Ovchinnikov Institute of Bioorganic Chemistry, Russian Academy of Sciences (Pushchino, Moscow Region, Russia). The animals were maintained at a 12 h light/dark cycle in a temperature- and humidity-controlled environment with free access to standard laboratory food and water. All experiments were conducted during the light phase (from 9 a.m. to 6 p.m.). The mice were randomly divided into three groups: Control group (administration of saline, *n* = 14), MPTP group (*n* = 12), and MPTP +Lf group (*n* = 12). MPTP (40 mg/kg, ABCR, AB456164) was administered once subcutaneously (s.c.) [7,32]. Control animals received 0.9% physiological saline instead of MPTP in an identical manner. Human Lf was isolated from the colostrum by preparative ion-exchange chromatography [33], followed by purification with affinity heparin-sepharose sorbent [34]. According to HPLC analysis [35], purity of the isolated protein was 97%. Human Lf (4.0 mg/animal), 0.4 mL in phosphate buffered saline (PBS; 0.1 M, pH 7.4), or PBS (vehicle) was administered intraperitoneally (i.p.; twice, 24 and 3 h before MPTP). Dosage of protein was based on the results of our previous experiments [24,31].

**Figure 1:**
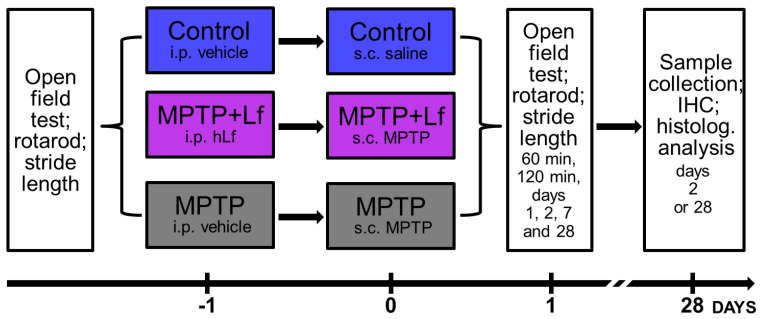
Experimental timeline. C57Bl/6 mice received an intraperitoneal injection of human Lf (i.p.; twice, 24 and 3 h before MPTP). MPTP (40 mg/kg) was administered once subcutaneously (s.c.). Behavioral and functional tests were performed prior to injections and 60 min, 120 min, 1, 2, 7, and 28 d after administration of MPTP/saline. On days 2 or 28, the animals were transcardially perfused; the brains were removed for immunohistochemical (IHC) analysis.

In all groups, behavioral and functional tests were performed prior to injections and 60 min, 120 min, 1, 2, 7, and 28 d after administration of MPTP/saline. When using multiple tests on the same group of animals, the tests were performed in the same order for all experimental groups. The open field test was always conducted first. The study design flowchart is displayed in Figure 1. The body weights of the mice were measured weekly throughout the experiment. In our previous study, it was shown that hLf administered i.p. had no impact on mouse motor performance in the open field test [31]. Therefore, to minimize the number of experimental animals, according to the principles of the 3Rs [36], the group treated with hLf alone was not included in our experiments. All experimental procedures were performed in accordance with the rules of the Ministry of Health of the Russian Federation (No. 267 of 19.06.2013) and the Local Ethics Committee for Biomedical Research of the National Research Center “Kurchatov Institute” (protocol No. 6 of 22.11.2017).

### 2.2. Changes in Body Weight

The body weights of the mice were measured on day −1 (Db) to serve as baseline before injections. Thereafter the animals’ weights were measured on each other day (Dt) until day 28. Change in body weight was determined by the following equation:

Change in body weight = (W at Dt − W at Db)/(W at Db) × 100%(1)

where W is the body weight of the animal.

### 2.3. Open Field Test

The open field test (OF) is a typical method used to study spontaneous motor activity and exploratory behaviors [37]. The open field was a round plastic arena (d = 120 cm, h = 45 cm). The central area (d = 60 cm) was designated as the center. Animals were individually placed into the center and allowed to freely explore OF for 5 min. The trajectories of animal movements were recorded using an automated video tracking system consisting of a Sony video camera (Japan) located 2.5 m above the arena and an EthoVision XT 8.5 video recording system (Noldus Information Technology, Wageningen, The Netherlands). The videos were analyzed using EthoVision XT 8.5 software. After each experimental session, the mice were carefully removed from the OF and returned to their home cage, and the inner walls and bottom of the arena were cleaned with 70% ethanol. The following parameters were recorded: distance traveled (total, central), number of rearings, latent period of exit from the central area, percent time spent in the center, and number of entries into the central area.

### 2.4. Rotarod

The rotarod test is one of the most commonly used tools for testing motor coordination and balance in mice [38]. The animals were briefly pre-trained on an automated five-lane rotarod unit (Ugo Basile, Italy, 3 cm diameter drums with grooves to improve grip) at a constant rotation speed of 30 rpm. In the test sessions, the latency of animals falling from the rod was measured as the time from the beginning of the trial until the mouse falls off onto the lever that stops the timer. The maximum trial length was 120 s. Each animal was tested twice, and the mean of the two trials was used for further analysis.

### 2.5. Stride Length

On the day of testing, animal fore and hind paws were coated with non-toxic paints of different colors. The mice were placed in a narrow passage (7 cm wide, 40 cm long, with walls of 10 cm height) lined with white paper and were allowed to walk freely. The animals were immediately put back into their home cage upon completion of the task. Stride lengths were determined by measuring the distance between each pawprint on the same side of the body (from the middle toe of the first pawprint to the heel of the second pawprint). Only strides made while continuously walking (without stops) were included in the analysis. The strides made at the beginning (7 cm) and the end (7 cm) of the passage were excluded because of velocity changes [39,40]. The stride length was measured in three animal steps, and the mean value was used in the analysis.

### 2.6. Sample Collection

On days 2 (these animals were randomly selected using a blind method) or 28 after MPTP/saline administration, the mice were anaesthetized by intramuscular injection of zoletil (Virbac Sante Animale, Chambray-lès-Tours, France) and rometar (Bioveta, Iva-novice na Hané, Czech Republic) in 0.9% NaCl (Dalkhimpharm, Khabarovsk, Russia), 0.1 mL per 10 g body weight. The animals were transcardially perfused with PBS and then with 4% paraformaldehyde in PBS. Perfusion was performed using an Ecoline ISM1090 peristaltic pump (Ismatec, Glattbrugg, Switzerland). The brains were removed from the skull and postfixed in 4% paraformaldehyde at 4 °C for 24 h. Coronal brain sections (40 μm) were cut through the striatum (+1.42 to −0.10 mm from bregma) and substantia nigra (−2.80 to −3.52 mm from bregma) on a Leica VT1200S vibratome (Leica, Nussloch, Germany). Every third section was collected to provide that the selected structure is represented in its entirety, but so that the same cells are not counted twice (which could happen if neighboring slices are analyzed). Anatomical landmarks were determined according to the atlas of the mouse brain [41].

### 2.7. Immunohistochemistry (IHC)

Tyrosine hydroxylase (TH) is the first and rate-limiting enzyme involved in the biosynthesis of catecholamines from tyrosine. For this reason, TH is considered as a useful marker of dopaminergic neurons [42,43]. For IHC analysis, free-floating sections were first rinsed (3 × 15 min) with 1 mL of 0.1 M PBS (pH 7.4). Endogenous peroxidase was blocked with 0.3% H_2_O_2_ (30 min). Then the sections were washed with PBS (3 × 15 min) and incubated in 10 mM citrate buffer, pH 6.0, for 10 min at 95 °C. For reduction of nonspecific staining and membrane permeabilization, the sections were preincubated with 2.5% normal goat serum (Abcam, ab7481) in 1.0% Triton X-100 (1% PBST) for 1 h at RT and washed three times with 0.2% PBST for 10 min. Then, the sections were incubated with primary anti-TH antibody (1:750; Abcam, ab112) in 0.2% PBST containing 0.01% sodium azide and 2.5% normal goat serum at 4 °C (ON) and then for 2 h at RT, washed in 0.2% PBST (3 × 15 min). The secondary goat anti-rabbit IgG H&L (HRP) (1:2000; Abcam, ab205718) in 0.2% PBST was applied (2 h at RT) followed by PBS rinses (3 × 15 min). Visualization was performed by incubation in 3,3′-diaminobenzidine (DAB substrate kit; Abcam, ab64238). The sections containing SN were counterstained with cresyl violet. The sections were then washed, dehydrated, cleared with xylene, and coverslipped using Mount-Quick mounting medium (Daido Sangyo, Saitama, Japan). To test the immunostaining specificity, control sections were processed in the same manner, but with the primary antibody omitted.

### 2.8. Histological Analysis

The sections immunostained for TH were imaged under a Zeiss Imager Z2 VivaTome light microscope (Carl Zeiss, Jena, Germany) and analyzed using AxioVision 4.8.2 software (Carl Zeiss, Jena, Germany). The sections from control and MPTP treated animals were coded and examined blind. TH-positive neurons and fibers were quantified.

#### 2.8.1. Counting of TH-Positive Cells in the Substantia Nigra

In each section, the region of interest was outlined, and TH+ cells in that region were selected and semi-automatically counted. The loss of DA-ergic neurons was determined by counting TH-positive cells in SNpc (5–7 representative sections from each mouse). Analysis was performed by counting TH+ neurons (phenotypic marker) and cresyl violet stained cells (structural marker) in both right and left SNpc in each representative section. The mean number of TH+ neurons was calculated for each animal. The neuronal counts are expressed as mean number of neurons per representative section [42].

#### 2.8.2. Measurement of TH-Positive Fibers Density in the Striatum

The content of TH+ fibers in the striatum was evaluated by measuring optical density (OD) of specific staining in five striatum sections from each mouse (four areas in each section) using ImageJ (Fiji version) software [44]. Background OD (measured in the cortex) was subtracted from the mean OD in the striatum [45]. OD in the striatum was standardized to OD in the cortex.

### 2.9. Statistical Analysis

The statistical analysis was performed using GraphPad Prizm 6.01 software (San Diego, CA, USA). The normality of data distribution was assessed with the Shapiro–Wilk test. The nonparametric Kruskal–Wallis ANOVA with *post hoc* Dunn’s test for multiple comparisons was employed. The differences within the group between the two test periods were evaluated using nonparametric Wilcoxon matched pairs test or Mann–Whitney U test as appropriate. Spearman’s rank correlation coefficient was used to examine the relationships between the parameters in behavioral tests and the numbers of TH-positive immunoreactive neurons and fibers. The data are presented as the median, quartiles, and min–max range. *p* values < 0.05 were considered to be significant.

## 3. Results

The MPTP-treated animals exhibited generalized tremor, piloerection, straub tail, rigidity, and hypokinesia soon after injection (data not shown). Pretreatment with hLf did not prevent these acute effects of MPTP. Dynamic assessment of locomotor and autonomic functions of mice treated with MPTP in the presence or absence of Lf showed that all types of extrapyramidal disorders were clearly manifested in ~2 min after the injection of neurotoxin. Low- and medium-amplitude generalized tremor appeared soon after injection and persisted for 30–40 min. Piloerection appeared in 2–3 min. Straub tail, rigidity, and hypokinesia appeared in 10–14 min.

### 3.1. Changes in Body Weight

**Figure 2:**
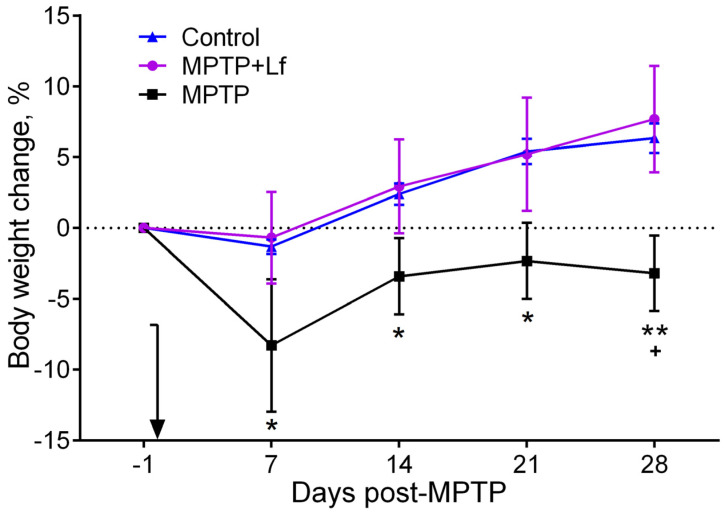
Body weight change (%) in the MPTP/saline-treated mice. The arrow indicates when MPTP/saline was administered. Values are presented as mean ± SEM. * *p* < 0.05, ** *p* < 0.01—compared to the Control group (saline-treated); + *p* < 0.05 compared to the MPTP + Lf group at an each time point (Kruskal–Wallis ANOVA followed by *post hoc* Dunn’s multiple comparisons test).

The body weight changes are summarized in Figure 2. On day 7 after treatments, body weight reduction was observed in all groups, although it was insignificant in the Control and MPTP + Lf groups. The body weight of animals in the MPTP group increased between days 7 and 14 and remained mostly constant between days 14 and 28. The percentage of body weight gain in the MPTP group was significantly lower compared with the Control group on day 7 (*p* = 0.049), day 14 (*p* = 0.030), day 21 (*p* = 0.018), day 28 (*p* = 0.008), and with MPTP + Lf group on day 28 (*p* = 0.026). The mice gradually gained weight from day 7 to day 28 in the Control and MPTP + Lf groups. By day 14, the body weights in both groups (Control and MPTP + Lf) began to exceed the baseline. There were no differences in body weight gain between MPTP + Lf and Control groups throughout the experimental period.

### 3.2. Rotarod

**Figure 3:**
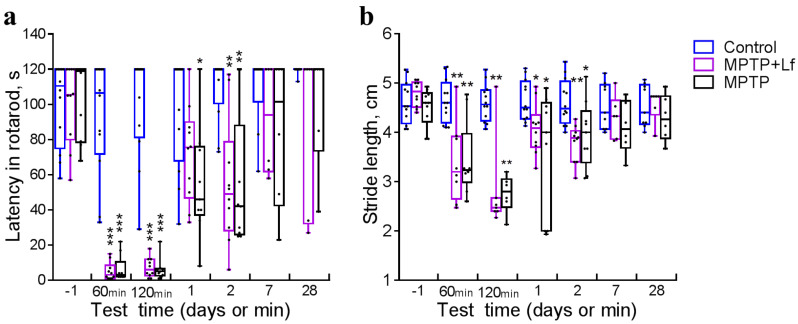
Latency in rotarod (**a**) and stride length (**b**) of mice after MPTP/saline administration. Each dot represents a single animal. *n* = 9–12 (from day −1 to day 2), *n* = 6–9 (days 7, 28) for each group. * *p* < 0.05, ** *p* < 0.01, *** *p* < 0.001 compared to the Control group (saline-treated) at each time point (Kruskal–Wallis ANOVA followed by *post hoc* Dunn’s multiple comparisons test).

We used the rotarod to detect the motor deficit. All mice used in this study showed learning of the rotarod test and reached a stable level of performance on the day before MPTP/saline injection (Control: 100.7 ± 6.8 s; MPTP + Lf: 100.7 ± 7.2 s; MPTP: 103.6 ± 6.9 s). The control mice maintained balance on the rotarod for practically the whole test (120 s). MPTP was shown to reduce the time spent on the rod at 60 min [H (2, *n* = 31) = 21.78, *p* < 0.0001] (MPTP + Lf: 5.1 ± 1.6 s; MPTP: 6.4 ± 2.5 s), 120 min [H (2, N = 31) = 22.11, *p* < 0.0001] (MPTP + Lf: 7.3 ± 1.8 s; MPTP: 6.2 ± 2.1 s), and 2 days [H (2, *n* = 31) = 13.97, *p* = 0.001] after treatment. However, on day 1, this parameter was significantly lower only in the MPTP (*p* = 0.015) but not in the MPTP + Lf group (*p* = 0.185) compared to the Control group [H (2, *n* = 31) = 7.489, *p* = 0.024]. The results of post hoc analysis are shown in Figure 3a. No significant differences were observed between both MPTP-treated groups and Control group in tests performed at 7 d [H (2, *n* = 21) = 2.062, *p* = 0.376] and 28 d [H (2, *n* = 21) = 1.835, *p* = 0.440] post-injection.

### 3.3. Stride Length

The day before MPTP/saline administration, no significant differences between the groups were observed (Figure 3b). The mean stride length in the Control group was almost constant throughout the experimental period. The acute MPTP intoxication induced a decrease in the mean stride length. In animals of the MPTP and MPTP + Lf groups, this parameter was reduced in comparison with the Control in 60 min [H (2, *n* = 29) = 12.33, *p* = 0.002] and 120 min [H (2, *n* = 25) = 14.17, *p* = 0.001] and on day 1 [H (2, *n* = 31) = 7.845, *p* = 0.019] and day 2 [H (2, *n* = 31) = 12.37, *p* = 0.002]. Nevertheless, Wilcoxon matched pairs test showed a significant increase in the mean stride length in the MPTP + Lf group (*p* = 0.031) one day after treatment. Recovery of stride length was observed in both the MPTP-treated groups on day 7 [H (2, *n* = 21) = 2.648, *p* = 0.2767] and day 28 [H (2, *n* = 21) = 1.684, *p* = 0.447].

### 3.4. Open Field Test

All mouse groups showed similar motor activity and exploratory behavior before MPTP/saline injection. Repeated OF testing led to time-dependent changes in the behavior of Control group animals. Fear-related behavior in response to novelty observed at the initial stages decreased, and motor activity decreased due to habituation.

**Figure 4:**
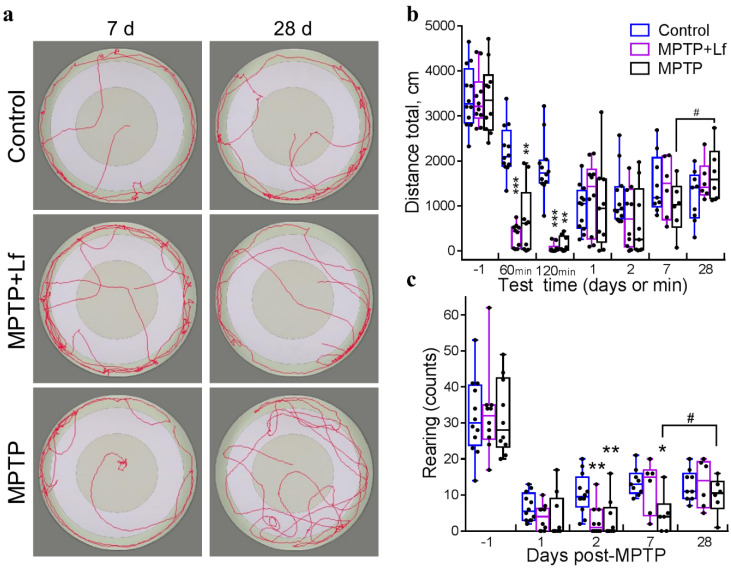
Behavior of mice in open field test after MPTP/saline administration. Representative five minute movement tracks of open field behavior by the mice on day 7 (**left**) and day 28 (**right**) (**a**). Total distance traveled (**b**). Number of rearings (**c**). Each dot represents a single animal. *n* = 9–12 (from day −1 to day 2), *n* = 6–9 (days 7, 28) for each group. * *p* < 0.05, ** *p* < 0.01, *** *p* < 0.001 compared to the Control group (saline-treated) at an each time point (Kruskal–Wallis ANOVA followed by *post hoc* Dunn’s multiple comparisons test); # *p* < 0.05 compared to the same group at the previous time point (Wilcoxon matched pairs test).

Acute MPTP intoxication reduced locomotor activity in mice. Representative 5-min tracks of OF behavior on days 7 and 28 are shown in Figure 4a (left and right panels, respectively). The total distance traveled in OF decreased in both MPTP and MPTP + Lf groups in 60 min [H (2, *n* = 31) = 19.12, *p* < 0.0001] and 120 min [H (2, *n* = 31) = 22.11, *p* < 0.0001] after treatment compared to the control (Figure 4b). At later terms, no differences in this parameter were observed between all three groups. Wilcoxon matched pairs test showed a significant increase in the total distance traveled in the MPTP group (*p* = 0.031) on day 28 compared to day 7. 

MPTP intoxication sharply suppressed behavioral activity on the day of treatment. Therefore, we did not compare mouse groups in 60 and 120 min after treatment by the following parameters: latent period of exit from the central area, percent time spent in the center, number of entries into the central area, and number of rearings. 

No differences between the groups were found in number of rearings [H (2, *n* = 31) = 4.263, *p* = 0.119]: 3.8 ± 1.1 in MPTP + Lf vs. 6.6 ± 1.1 in Control (*p* = 0.242) and 4.0 ± 2.1 in MPTP (*p* = 0.113) on day 1 (Figure 4c). On day 2, the number of rearings in both MPTP-treated groups was lower than in the Control group [H (2, *n* = 31) = 12.180, *p* = 0.002; *p* = 0.007 for MPTP, *p* = 0.006 for MPTP + Lf], but on day 7, this parameter was significantly below the control only in the MPTP group [H (2, *n* = 21) = 6.235, *p* = 0.038; *p* = 0.041 for MPTP, *p* > 0.999 for MPTP + Lf]. Significant differences were observed in number of rearings in the MPTP group on days 28 and 7 (*p* = 0.031, Wilcoxon matched pairs test). No differences between the groups were detected on day 28.

**Figure 5:**
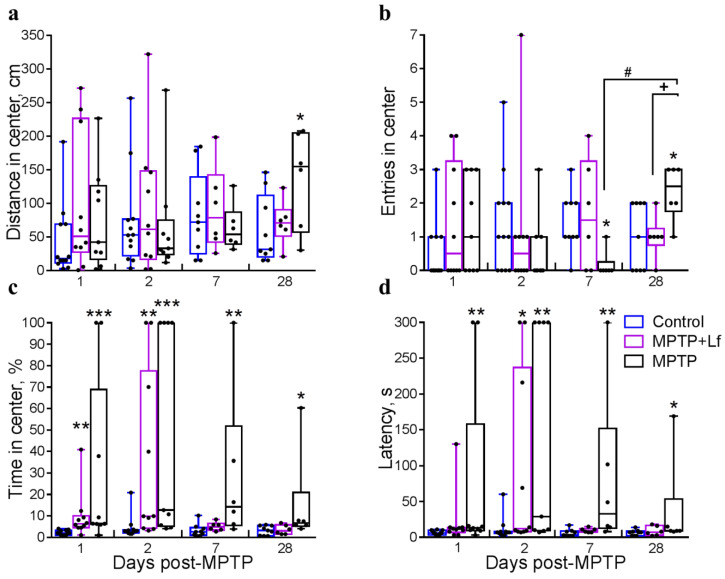
Behavior of mice in the center of the open field test (**a**–**d**) after MPTP/saline administration. Distance traveled in the center of the field (**a**). Number of entries into the central area (**b**). Percent time spent in center (**c**). Latency to exit from the central area (**d**). Each dot represents a single animal. *n* = 9–12 (days 1, 2), *n* = 6–9 (days 7, 28) for each group. * *p* < 0.05, ** *p* < 0.01, *** *p* < 0.001 compared to the Control group (saline-treated); + *p* < 0.05 compared to the MPTP + Lf group at each time point (Kruskal–Wallis ANOVA followed by *post hoc* Dunn’s multiple comparisons test); # *p* < 0.05 compared to the same group at the previous time point (Wilcoxon matched pairs test).

We revealed no differences in the central distance traveled between the MPTP + Lf and Control groups over the experimental period except 120 min after MPTP administration. In this time point, mice of both MPTP-treated groups travelled shorter distance in the center than the controls [H (2, *n* = 31) = 12.93, *p* = 0.002]: 33.0 ± 12.5 cm in MPTP + Lf vs. 129.1 ± 21.3 cm in Control (*p* = 0.002) and 41.4 ± 18.6 cm in MPTP (*p* = 0.012). However, the MPTP group exhibited a significant increase in this parameter compared to the Control group on day 28 [H (2, *n* = 21) = 5.240, *p* = 0.068; *p* = 0.046 for MPTP, *p* = 0.972 for MPTP + Lf] (Figure 5a). The MPTP group showed a tendency to an increase in the distance traveled in the center on day 28 compared to day 7 (Wilcoxon matched pairs test, *p* = 0.063).

No differences between the groups were found by the number of entries into the center on day 1 [H (2, *n* = 31) = 2.168, *p* = 0.338]: 1.4 ± 0.5 in MPTP + Lf vs. 0.5 ± 0.3 in Control (*p* = 0.428) and 1.3 ± 0.5 in MPTP (*p* = 0.418); day 2 [H (2, *n* = 31) = 2.851, *p* = 0.240; *p* = 0.203 for MPTP, *p* = 0.551 for MPTP + Lf] (Figure 5b). On day 7, the number of entries into the center in the MPTP group was significantly lower than in the Control group [H (2, *n* = 21) = 6.792, *p* = 0.029; *p* = 0.030 for MPTP, *p* > 0.999—MPTP + Lf] and MPTP + Lf group, though the difference in the latter case did not reach statistical significance (*p* = 0.069). On the contrary, on day 28 the number of entries into the center in the MPTP group was significantly higher [H (2, *n* = 21) = 6.926, *p* = 0.023] than in the Control (*p* = 0.035) and MPTP + Lf (*p* = 0.049) groups. Wilcoxon matched pairs test showed a significant increase in this parameter in the MPTP group (*p* = 0.031) on day 28 compared to day 7. We found no differences in the number of entries into the center between the MPTP + Lf and Control groups throughout the experimental period.

The MPTP-treated mice spent more time in the center of OF than the control on day 1 [H (2, *n* = 31) = 15.320, *p* = 0.001]: 9.9 ± 3.6% in MPTP + Lf vs. 2.2 ± 0.3% in Control (*p* = 0.004) and 30.4 ± 13.6% in MPTP (*p* = 0.001); day 2 [H (2, *n* = 31) = 15.320, *p* = 0.001; *p* = 0.001 for MPTP, *p* = 0.005 for MPTP + Lf] (Figure 5c). Nevertheless, alone, the MPTP group exhibited a significant increase in this parameter compared to the Control group on day 7 [H (2, *n* = 21) = 9.572, *p* = 0.004; *p* = 0.004 for MPTP, *p* = 0.382 for MPTP + Lf] and day 28 [H (2, *n* = 21) = 7.494, *p* = 0.017; *p* = 0.019 for MPTP, *p* > 0.999 for MPTP + Lf]. The same tendency was verified compared to the MPTP + Lf group (*p* = 0.065) on day 28.

Finally, the MPTP group showed higher latency to exit from the central area than the Control group over the experimental period after MPTP/saline administration (Figure 5d): on day 1 [H (2, *n* = 31) = 9.411, *p* = 0.009; *p* = 0.007 for MPTP, *p* = 0.072 for MPTP + Lf], day 2 [H (2, *n* = 31) = 12.160, *p* = 0.002; *p* = 0.003 for MPTP, *p* = 0.017 for MPTP + Lf], day 7 [H (2, *n* = 21) = 9.685, *p* = 0.004; *p* = 0.004 for MPTP, *p* = 0.376 for MPTP + Lf], and day 28 [H (2, *n* = 21) = 5.199, *p* = 0.069; *p* = 0.046 for MPTP, *p* = 0.933 for MPTP + Lf]. The same trend was verified compared to the MPTP + Lf group (*p* = 0.072) on day 1. The MPTP + Lf and Control groups showed similar latency to exit from the central area over the experimental period, except day 2.

The parameters “percent time spent in the center” and “latent period of exit from the central area” complement each other and, together with the number of visits to the center and distance traveled in the central area, characterize the behavioral strategy of the mouse. It is known that different behavioral parameters recover at different rates. The behavior in the OF test reflects the balance between fear, exploratory behavior, and the animal’s motor capabilities. Gradual recovery of motor and exploratory activity occurred in MPTP-treated mice after their strongest suppression, which was observed at 60 and 120 min after administration of the neurotoxin. According to the results of the rotarod and the stride length, it is clear that the motor deficit in these animals disappeared by day 7. However, in the MPTP group, the effect of the neurotoxin was observed for a longer time than motor deficiency. Many behavioral parameters did not recover even by day 28. The MPTP + Lf animals exhibited the recovery of all behavioral recorded parameters on day 7.

### 3.5. Histological Analysis

**Figure 6:**
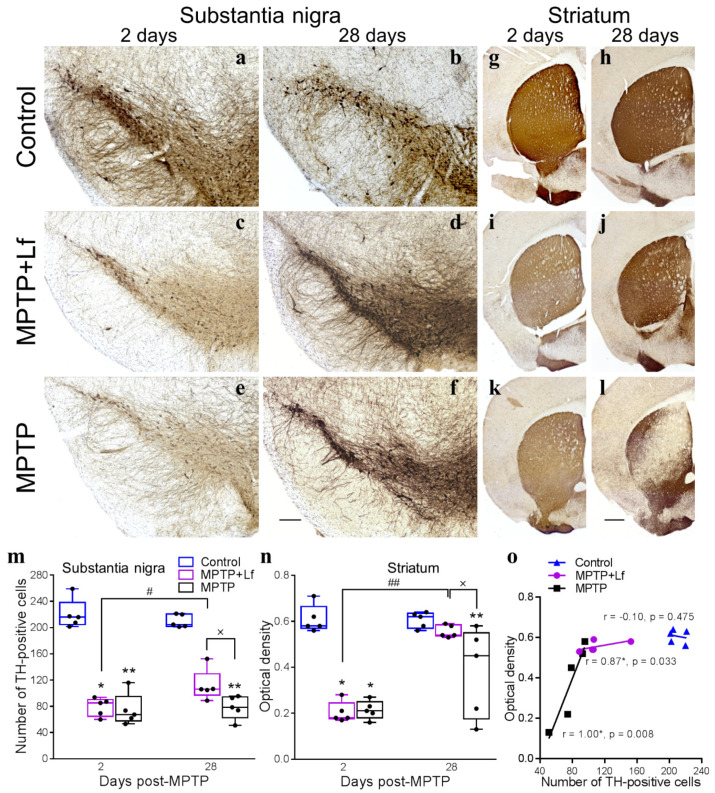
Effects of Lf on MPTP-induced neurodegeneration. Representative photomicrographs of SNpc (**a**–**f**) and striatum (**g**–**l**) sections immunostained for TH. Number of TH-positive neurons in SNpc; *n* = 5–7 representative sections from each mouse (**m**). Each dot represents a single animal. Striatal TH-positive optical density; *n* = 5 sections from each mouse (**n**). Correlation between the SNpc TH-positive neuronal counts and the striatal TH-positive optical density on day 28 (**o**). Scale bars = 200 μm (**a**–**f**), 500 μm (**g**–**l**). * *p* < 0.05, ** *p* < 0.01 compared to the Control group (saline-treated) at an each time point (Kruskal–Wallis ANOVA followed by *post hoc* Dunn’s multiple comparisons test); # *p* < 0.05, ## *p* < 0.01 compared to the same group at the previous time point; ^x^ *p* < 0.05 (Mann–Whitney U test).

Administration of MPTP induced loss of dopaminergic neurons in the SNpc (Figure 6a,c,e) and dopaminergic fibers in the striatum (Figure 6g,i,k) on day 2 after treatment. Histological analysis revealed a significant decrease in the number of TH-positive cells in the SNpc [H (2, *n* = 15) = 9.620, *p* = 0.002; *p* = 0.008–PTP, *p* = 0.033–MPTP + Lf] (Figure 6m) and reduction of optical density TH-specific staining in the striatum [H (2, *n* = 15) = 9.506, *p* = 0.002; *p* = 0.024–MPTP, *p* = 0.010–MPTP + Lf] (Figure 6n) in the MPTP-treated mice compared to the control.

The loss of TH-positive neurons in the SNpc was irreversible in MPTP group. According to the Kruskal–Wallis ANOVA, the number of TH-positive cells in this group remained significantly lower than in the control on day 28 [H (2, *n* = 15) = 11.580, *p* < 0.0001; *p* = 0.001–MPTP, *p* = 0.113–MPTP + Lf]. In contrast, the MPTP + Lf group exhibited a significant increase in this parameter on day 28 (Mann–Whitney test: U = 1.000, *p* = 0.016) (Figure 6b,d,f,m). The number of TH-positive cells in the MPTP + Lf group was significantly higher than in the MPTP group on day 28 (Mann–Whitney test: U = 2.000, *p* = 0.032) (Figure 6m).

Regarding TH-positive fibers in the striatum, the MPTP group showed a significant decrease in optical density of TH-specific staining compared to the Control group on day 28 [H (2, *n* = 15) = 8.072, *p* = 0.009; *p* = 0.009–MPTP, *p* = 0.311–MPTP + Lf]. Importantly, the MPTP + Lf group exhibited a significant increase in this parameter on day 28 (Mann–Whitney test: U = 0.00, *p* = 0.008) (Figure 6h,j,l,n). The optical density of TH-positive fibers in the MPTP + Lf group was significantly higher than in the MPTP group on day 28 (Mann–Whitney test: U = 3.500, *p* = 0.036) (Figure 6n). No differences in number of TH-positive cells in the SNpc or TH-positive fibers in the striatum between the MPTP + Lf and the Control groups were detected on day 28.

Our analyses showed that the positive correlation between the number of TH+ neurons in the SNpc and OD of TH+ fibers in the striatum existed in both MPTP-treated groups on day 28 (Figure 6o). However, no correlation was observed in the Control group.

### 3.6. Correlation between the Number of Rearings in OF Test and Number of TH-Positive Immunoreactive Neurons and Fibers

**Figure 7:**
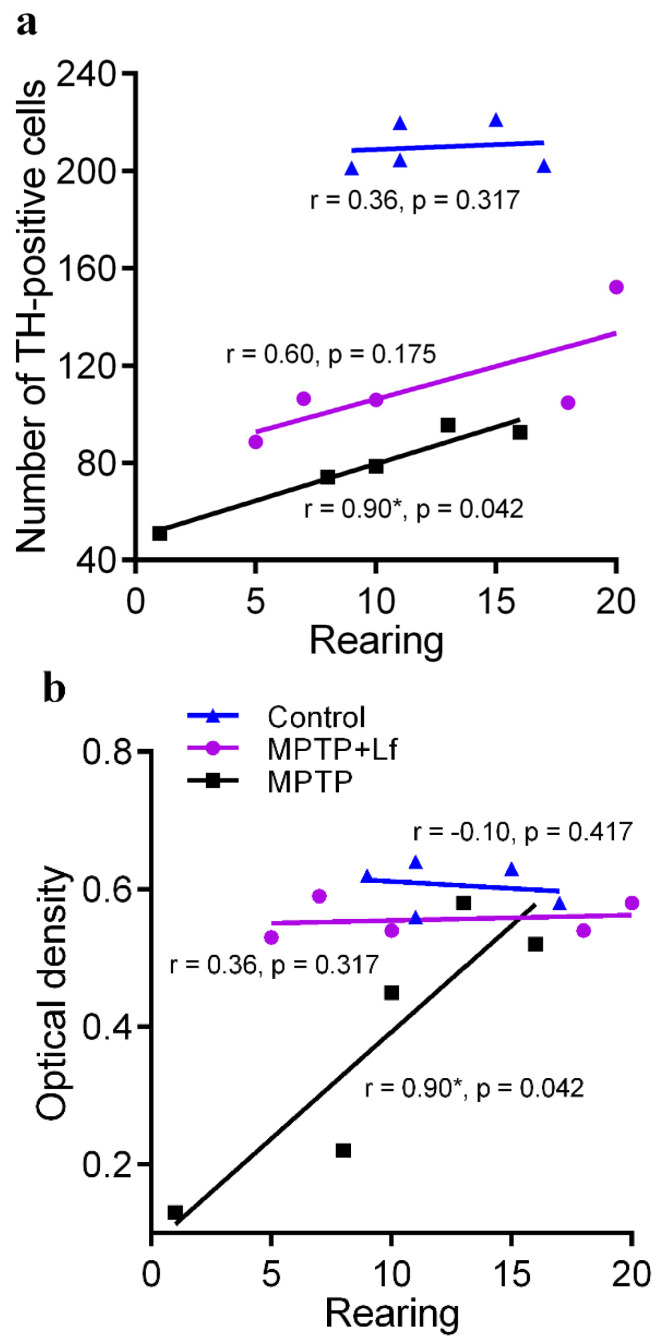
Relationship between the number of rearings in open field and the number of TH-positive neurons (**a**) and the optical density of TH-positive fibers (**b**) of the MPTP/saline-treated mice on day 28. * significant correlation (Spearman’s rank correlation).

In the MPTP group, a positive correlation was observed between the number of TH+ neurons in the SNpc or the optical density of TH-immunoreactive fibers in the striatum on day 28 and the number of rearings in OF on the same day (Figure 7a,b). No such correlation was observed in the MPTP + Lf and Control groups.

## 4. Discussion

Neurodegenerative alterations in PD are accompanied by behavioral deficits, especially in motor activity. Studying the dynamics of these changes in animal model of PD is of particular interest for investigation of the relationship between neuronal degeneration, recovery processes, and manifested behavior. In order to determine whether pretreatment with hLf alleviates degenerative changes in the nigrostriatal system and associated behavioral disturbances caused by MPTP administration to adult C57Bl/6 mice, extensive analysis of animal behavior was performed.

The present data showed that there were no differences in body weight gain between MPTP + Lf and Control groups throughout the experimental period (Figure 2). These mice gradually gained weight from day 7 to day 28. However, the weight’s dynamic was different in the MPTP group. The body weight of animals in the MPTP group decreased sharply on day 7 and did not recover by day 28. Thus, Lf had a protective effect on body weight gain, since it was administered before the neurotoxin.

The acute MPTP administration (single dose, 40 mg/kg, s.c.) induced long-term behavioral deficit. The MPTP intoxication significantly decreased spontaneous motor activity and exploratory behavior on the day of administration. In the open field test, a serious reduction of all analyzed parameters (distance traveled, number of rearings, number of entries into the center, etc.) was observed 2 h after MPTP injection. This is consistent with published data [32,46] on reduction of locomotor activity when a similar scheme of MPTP administration was used. Pretreatment with hLf did not prevent these acute effects of MPTP.

The recovery process differed between the groups. In the MPTP group, the number of rearings was lower than in the Control group on days 2 and 7. Then, this group demonstrated a significant increase in the total distance traveled, the number of rearings, and number of entries into the center on day 28 compared to day 7. The total distance traveled and the number of rearings recovered to the control levels on days 1 and 28, respectively, whereas the MPTP group continued to differ from the Control group for most of the behavioral parameters even on day 28. Rosa et al. [46] demonstrated that acute MPTP administration induced long-term alterations (30–45 d post injection) in C57BL/6 mice motor performance paradigms. Previously it was shown that MPTP (2 × 40 mg/kg, s.c.) considerably reduced spontaneous motor activity, since the mice demonstrated as low an activity level at three weeks as at 12 weeks after treatment [47]. Previous studies also showed deficits in rearing behavior in MPTP-treated mice [48,49,50], and it was even heavier than deficits in locomotor activity. Schwarting et al. [49] demonstrated that a significant correlation existed between the content of dopamine in the striatum and rearing when animals had recovered in the parameters of locomotion. Therefore, the measure of rearing behavior seems to be a sensitive index of MPTP-generated damage in mice [49]. Dopamine in the neostriatum is thought to play a critical role as an interface between motivation and action [51].

The present data showed that the Lf-pretreated mice had completely recovered on day 7, some parameters (total and central distance traveled, number of entries into the center) returned to normal (control) levels on the next day after the MPTP-treatment (Figure 4 and Figure 5). The Lf-pretreated animals, likewise the control mice, exhibited the constancy of all behavioral recorded parameters on days 7 and 28. The present results suggest that hLf pretreatment stimulates rapid recovery of the MPTP-disturbed OF behavior. This is consistent with Xu et al. [16], who showed that injection of Lf (i.p., 4 mg/kg × 7 d) could effectively improve the movement and behavioral disorders caused by MPTP in six-month-old C57Bl/6 mice. Liu et al. [52] also demonstrated that Lf pretreatment (intragastric gavage, in various doses; 2 d before and 5 d during MPTP exposure) alleviated MPTP effect in the pole test on the day after the last introduction.

The mice treated with MPTP exhibited considerable impairment of motor coordination (the rotarod test) and decrease in the mean stride length at 60 min, 120 min, day 1, and day 2 after injection. Pretreatment with hLf did not prevent these acute effects of MPTP. Recovery of these parameters was observed in both MPTP-treated groups on day 7. These data correlate with findings obtained earlier. Rosa et al. [46] found no differences between MPTP and Control groups either in the rotarod test (on days 14, 37, and 52) or in the stride length analysis (on days 7, 30, and 45). Thereby, our results indicated that MPTP-induced motor deficit (the functional component of behavior) recovered much faster than exploratory activity, and Lf had a positive effect on the restoration process of the latter (Figure 4 and Figure 5).

In the present study, MPTP induced a loss of dopaminergic neurons in the SNpc and dopaminergic fibers in the striatum. On day 2, the number of TH-positive cells was decreased to 34% and 36%, and the OD of TH-positive fibers was reduced to 35% and 33% of the control in groups MPTP and MPTP + Lf, respectively (Figure 6). These disturbances were irreversible in the MPTP group, although the OD of TH-positive fibers in the striatum increased on day 28. Previously, it was shown [53] that MPTP treatment in a single dose of 40 mg/kg induced substantial loss of striatal dopaminergic terminals and TH-immunoreactive neurons in the substantia nigra. Densitometry analysis showed a significant reduction in the content of TH-positive fibers in mouse striatum on day 52 after MPTP injection in the same treatment regime [46]. Fredriksson and Archer [48] found a decrease in dopamine content in the striatum of mice in three weeks and three months after repeated injections of MPTP in different doses. MPTP administered in a series of injections (i.p., 20 mg/kg × 4) at 2 h intervals resulted in 60–70% loss of nigrostriatal neurons and 80–90% depletion of striatal DA levels measured 7 d after treatment [42,54]. Furthermore, Jakowec et al. [54] revealed no significant changes in the number of TH+ neurons in SNpc on days 30 and 60 after MPTP administration, but the striatal level of TH protein increased through days 30, 60, and 90, although remained below the initial level. Depboyli et al. [55] demonstrated that the loss of TH+ neurons in the SN was progressive and irreversible, but the decrease of TH+ fibers and DA levels in the striatum partially recovered by day 28 after MPTP injections (i.p., 30 mg/kg × 5, at 24 h intervals). Xu et al. [16] and Liu et al. [52] found that Lf substantially reduced the MPTP-caused loss of DA neurons in SN, nerve fibers, and DA depletion in the striatum. In both works, Lf was administered for seven consecutive days (either after or before and after the MPTP), and histological as well as biochemical analyzes were performed at only one time point. In our study, Lf was administered only twice and strictly before the neurotoxin administration. The present data showed that the time course of the nigrostriatal system recovery was observed in the hLf-pretreatment mice. The number of TH-positive cells and the OD of TH-positive fibers were increased to 53% and 92% of the control, respectively (Figure 6).

The neuronal dysfunction precedes neuronal death. MPTP can cause a loss of TH-immunoreactivity without necessarily destroying the neuron [42]. Xu et al. [56] demonstrated that MPTP significantly decreased TH gene expression. In the process of neurodegeneration, fiber loss precedes actual neuronal death [57], and MPTP intoxication elicits spontaneous long-term compensatory sprouting in mice [58]. The functional recovery effect may be caused by increased DA turnover in the remaining DAergic neurons or by increased number of dopaminergic receptors in the post-synaptic cells. Kolacheva and Ugrumov [59] showed that one of the mechanisms of neuroplasticity is a change in the TH activity for degeneration of nigrostriatal DAergic neurons and for partial functional recovery of the survived neurons. Furthermore, it is possible that new neurons are connected to TH production by changing own phenotype [60]. Lf pretreatment probably led to recovery by inducing one or more of these processes. It was previously shown that the half-life of hLf injected into the rat caudal vein is 10 min, and after 3 h, 10% protein remains in the circulation [61]. We can assume that Lf triggers a cascade of long-term events in the cell that help it to survive during acute neurotoxic exposure and subsequently restore its functional activity. These can be some mechanisms mediated by changes in gene expression [27] and, as a consequence, by long-term changes in cells exposed to both Lf and neurotoxin. Chen et al. [27] showed that Lf influenced above 10 genes involved in the brain-derived neurotrophic factor (BDNF) signaling pathway. BDNF participates in the survival of neurons and the promotion of growth and differentiation of new neurons and synapses.

The mechanism of Lf action is not yet clear enough. However, previous studies showed that Lf inhibits MPTP-induced oxidative stress and neuroinflammation [16,52] and protects against iron dysregulation [52] and mitochondrial dysfunction [62]. 

In the present study, we observed correlations between the rearing behavior in OF on the final day of testing (Figure 7) and the neural damage on day 28 after MPTP treatment. These relationships were found only in the MPTP group. Previous study had indicated a high correlation of the deficit in rotarod performance with the loss of striatal TH immunoreactivity [63]. Schwarting et al. [49] showed that the deficits in rearing were related to the degree of striatal DA depletion in the C57Bl/6 mice, both acutely after lesion and after four days. Our analyses showed that the positive correlation between the number of TH+ neurons in the SNpc and the OD of TH+ fibers in the striatum existed in both MPTP-treated groups on day 28. Based on our data, we can conclude that hLf had a protective effect and facilitated recovery of motor functions, exploratory behavior of mice, and functional activity of TH+ cells in the nigrostriatal system after acute MPTP exposure. The results of this work elucidate the role of lactoferrin in protective and compensatory mechanisms and provide the basis for potential use of this protein in the treatment of human neurodegenerative diseases.

## 5. Conclusions

Our data suggest that hLf pretreatment led to a significant alleviation in MPTP toxicity. This was manifested in less pronounced body weight loss (both in the acute and adaptive phases), improved motor functions and exploratory behavior, partial recovery of the number of TH-positive cells in SN, and TH-positive fibers in the striatum. We found that hLf pretreatment had a positive effect on the dynamics of recovery of mouse nigrostriatal system after acute MPTP exposure. Low dose hLf before the neurotoxin administration provided the protection and probably stimulates neuroregeneration under conditions of MPTP toxicity in our model. Further research is needed to determine exactly what the compensatory mechanisms are activated by this protein.
